# Caspases in Alzheimer’s Disease: Mechanism of Activation, Role, and Potential Treatment

**DOI:** 10.1007/s12035-023-03847-1

**Published:** 2023-12-23

**Authors:** Piotr Wójcik, Michał K. Jastrzębski, Agata Zięba, Dariusz Matosiuk, Agnieszka A. Kaczor

**Affiliations:** 1 Department of Synthesis and Chemical Technology of Pharmaceutical Substances with Computer Modeling Laboratory, Faculty of Pharmacy, Medical University of Lublin, 4A Chodzki St., 20093 Lublin, Poland; 2 School of Pharmacy, University of Eastern Finland, Yliopistonranta 1, P.O. Box 1627, 70211 Kuopio, Finland

**Keywords:** Alzheimer’s disease, Neurons, Caspases, Apoptosis

## Abstract

With the aging of the population, treatment of conditions emerging in old age, such as neurodegenerative disorders, has become a major medical challenge. Of these, Alzheimer’s disease, leading to cognitive dysfunction, is of particular interest. Neuronal loss plays an important role in the pathophysiology of this condition, and over the years, a great effort has been made to determine the role of various factors in this process. Unfortunately, until now, the exact pathomechanism of this condition remains unknown. However, the most popular theories associate AD with abnormalities in the Tau and β-amyloid (Aβ) proteins, which lead to their deposition and result in neuronal death. Neurons, like all cells, die in a variety of ways, among which pyroptosis, apoptosis, and necroptosis are associated with the activation of various caspases. It is worth mentioning that Tau and Aβ proteins are considered to be one of the caspase activators, leading to cell death. Moreover, the protease activity of caspases influences both of the previously mentioned proteins, Tau and Aβ, converting them into more toxic derivatives. Due to the variety of ways caspases impact the development of AD, drugs targeting caspases could potentially be useful in the treatment of this condition. Therefore, there is a constant need to search for novel caspase inhibitors and evaluate them in preclinical and clinical trials.

**Graphical Abstract:**

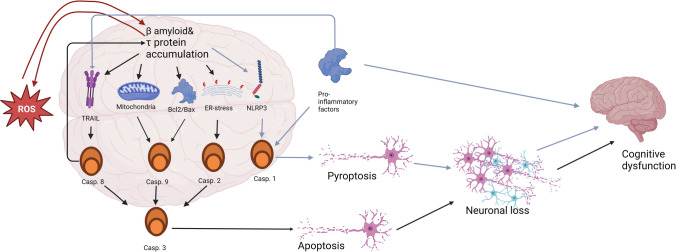

## Caspases

Caspases belong to the group of proteolytic enzymes, cysteine aspartate proteases, or more specifically, cysteine proteases with strict specificity for the aspartate residue at the P1 (N-terminal) position of the substrate. Various representatives of the family have different substrates (they recognize distinct amino acid residues adjacent to aspartic acid). Based on biochemical studies, two subfamilies of kinases associated with specific biological processes have been distinguished: kinases responsible for processing pro-inflammatory cytokines (cytokine activator subfamily 1, 4, 5) and kinases associated with programmed cell death (apoptotic subfamily)—in this subfamily, an additional distinction is made between apoptosis-initiating caspases (2, 8, 9, 10) and executioner caspases (3, 6, 7) [1]. An exception in this classification is caspase-14, whose activation is associated with terminal epidermal differentiation [2].

### Structure of Caspases

The subunits of each catalytic domain are αβα sandwiches, folded into a compact cylinder with six-stranded β-sheets in the center surrounded by five helices placed on opposite sides of the plane formed by the β-sheets. Despite the presence of additional strands in some structures, the overall arrangement remains intact. The catalytic dimer contains monomers arranged according to a double symmetry, and the two active sites are located at opposite ends of the dimer. The cleaved C-terminus of the large subunit and the N-terminus of the small subunit are located close to each other [3]. This configuration leads to the creation of the structure in which the two subunits of the active caspase molecule are formed into active caspase from different subunits of each zymogen via a domain-swapping model [4, 5]. This model assumes that the conformation of the zymogen is extremely close to the active conformation. What seems to be even more important is that each subunit of the active molecule is derived from a proenzyme and that cleavage of the linker between domains changes the orientation of the subunits [3].

### Features and Activation

Caspases are synthesized as inactive proenzymes (zymogens), composed of one large and one small subunit and a prodomain. Crystallographic studies of caspase-1 and caspase-3 indicate that the active enzyme is a heterotetramer, containing two small and one large subunit each. Phylogenetic analysis of caspases reveals the existence of three subfamilies: the ICE (Interleukin-1ß-converting enzyme) subfamily, which includes caspase-1, caspase-4, and caspase-5; the CED-3 (Caenorhabitis elegans cell death protein), CPP32 (32-kDa cysteine protease) subfamily, which includes caspase-3, caspase-6, caspase-7, caspase-8, caspase-9, and caspase-10; and the ICH-1 subfamily Nedd2 [6]. All caspases contain a pentapeptide in the active site with the general structure QACXG (where X is R, Q, or G). The amino acids Cys-285 and His-237 involved in catalysis and those involved in the formation of the P-carboxylate binding pocket in caspase-1 (Arg-179, Gln-283, Arg-341, and Ser-347) are conserved in the vast majority of caspase subtypes [1]. Studies such as those with caspase-1^−/−^ and caspase-3^−/−^ mice suggest that not all caspases are required for cell death, and some subtypes are more important than others. The key role of a particular caspase in apoptosis has often been inferred from its overexpression resulting in the induction of this process. However, such overexpression can lead the caspase to cleave substrates it does not normally recognize [6]. Some caspases contain only a short prodomain (caspase-3, caspase-6, and caspase-7), while others contain long prodomains (caspase-1, caspase-2, caspase-4, caspase-5, caspase-8, caspase-9, and caspase-10) [7]. The main determinant of specificity that distinguishes functional caspase groups from each other is the nature of the S4 pocket. Cytokine activators prefer aromatic residues (Trp or Tyr) in S4, apical caspase-8 and caspase-9 prefer hydrophobic residues such as Leu and Ile, and the executive caspases, caspase-3 and caspase-7, prefer Asp in this position. These preferences are partly responsible for the specificity of caspases toward natural substrates [3, 8].

### *Action and Catalysis*

Caspases show maximum activity at neutral pH (6.8 to 7.2), under reducing conditions, and at cytosolic ionic strength [9, 10]. At neutral pH, activated cysteine (Cys) would be polarized by catalytic histidine. This suggests that His237 is not directly involved in generating the catalytic nucleophile Cys but rather is important for protonating the α-amine leaving group and generating the nucleophilic water molecule required for deacylation [3].

**Fig. 1:**
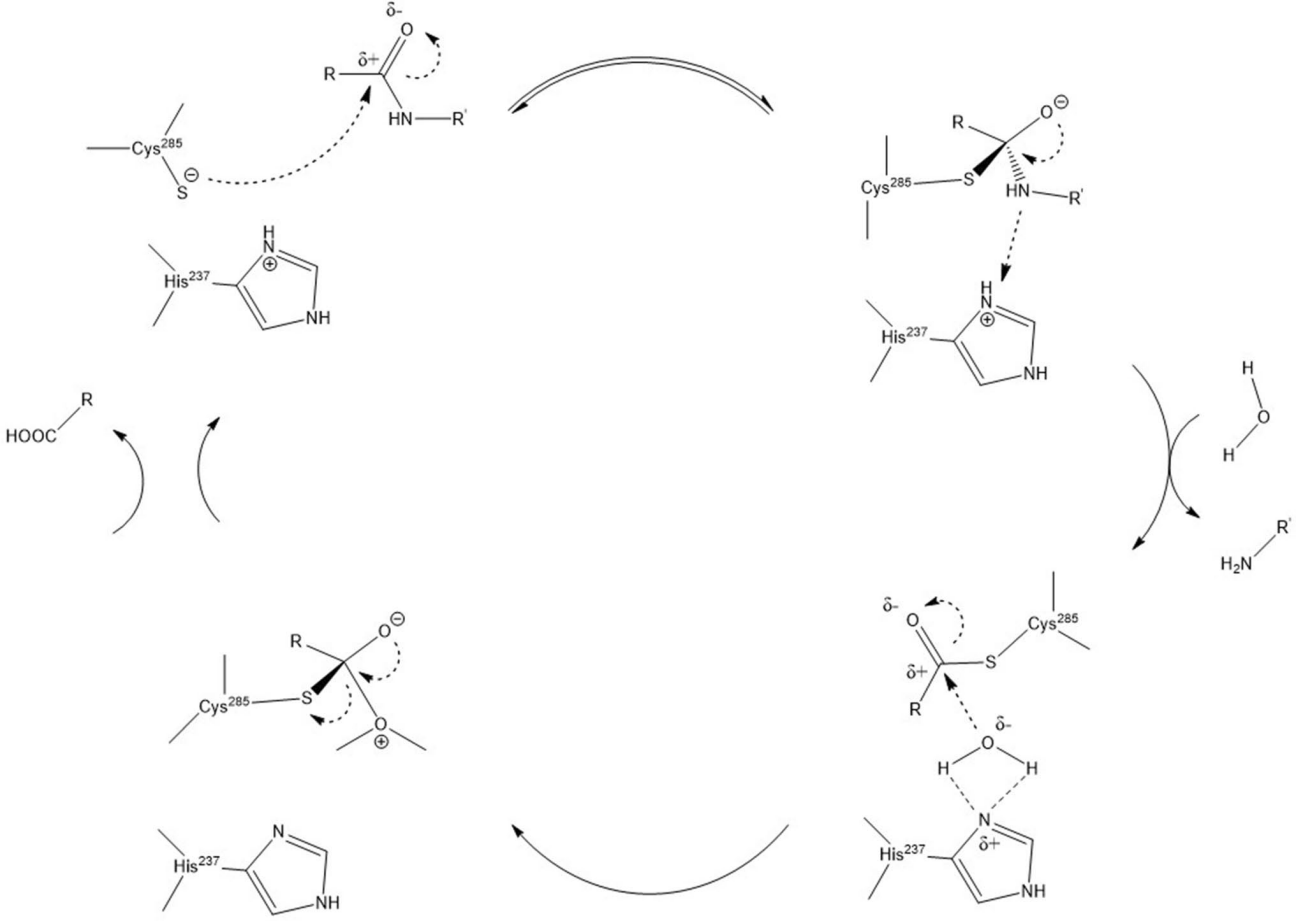
Catalytic mechanism of caspases

Caspases show strict specificity for the aspartate residue in the S1 substrate pocket. The S1 pocket is formed by the cooperation of at least three residues (Arg179, Gln283, and Arg341), which form hydrogen bonds with the carboxylate group of aspartate P1. This tightly controlled environment prevents other amino acids, such as glutamate, from selectively binding to the S1 site. As with other cysteine and serine proteases, the role of the catalytic site is to force the formation of a tetrahedral intermediate. Once the substrate is bound, the NH backbone of Gly238 and the Cys285 forming the oxyanionic hole donate the hydrogen bond to the carbonyl oxygen, thereby polarizing the carbonyl group of the scintillation bond. The nucleophilic Cys285 can now attack the electrophilic carbon. During or before this, the proton of the thiol group can be transferred to the neighboring His237. This proton can now act as a catalyst by protonating the amino group leaving the P1′ residue. During deacylation, His237 can polarize the water molecule needed to complete hydrolysis and form a second tetrahedral intermediate (Fig. 1) [11].

### Role of Caspases in Cell Death

Caspases are well-known for their role in apoptosis. Apoptosis can be induced by intracellular events, such as the accumulation of DNA damage and oxidative stress, leading to the activation of the intrinsic apoptosis pathway and caspase 9. It can also be induced by the accumulation of misfolded, abnormal, or oxidized proteins in the endoplasmic reticulum (ER), activating the ER stress-induced pathway and caspase 2. Additionally, extracellular regulators can induce apoptosis, such as the extrinsic pathway, which leads to the activation of initiator caspase 8. These pathways occur simultaneously, with one pathway activating others [12].

#### Extrinsic Apoptosis Pathway

**Fig. 2:**
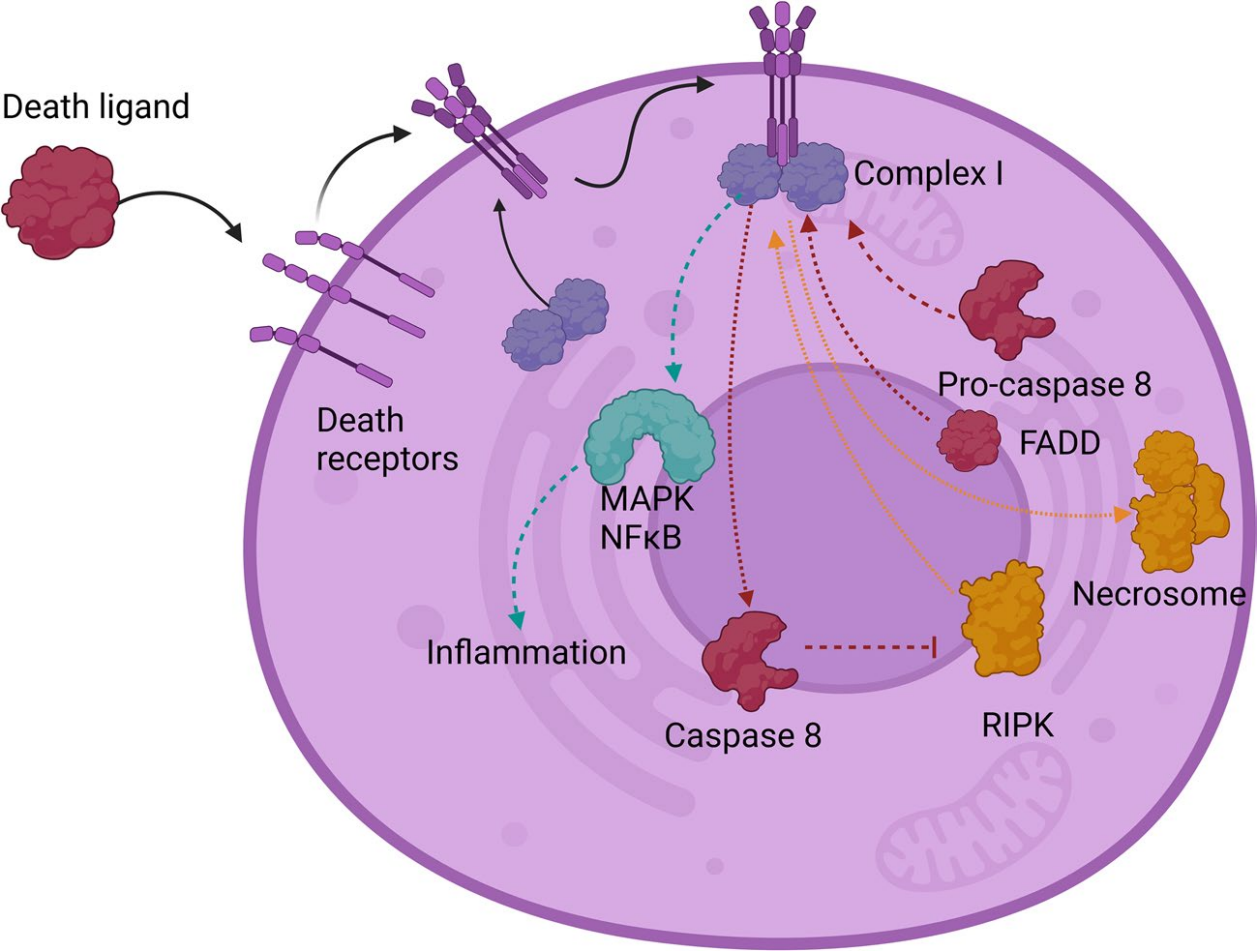
Different effects of death receptor activation

The extrinsic pathway is activated by the interaction between death ligands and death receptors (Fig. 2). Tumor necrosis factor receptors (TNFRs), TNF-related apoptosis-inducing ligand receptors (TRAIL-Rs), and cluster of differentiation 95 (CD 95) are well-known death receptors characterized by the presence of a highly conserved structural fold called the death domain [12]. Activation of these receptors leads to trimerization of the death domains [13]. In the case of TNFRs, tumor necrosis factor receptor type 1–associated DEATH domain protein (TRADD) and receptor-interacting serine/threonine-protein kinase 1 (RIPK1) proteins attach to the death domains, forming complex I. Subsequently, cellular inhibitor of apoptosis protein (cIAPs) and (TNF receptor–associated factors) TRAF2/3/5 are recruited to complex 1, mediating the ubiquitination of RIPK1, and stabilizing the complex. Complex 1 formation can lead to nuclear factor kappa-light-chain-enhancer of activated B cells (NFκB) and mitogen-activated protein kinase (MAPK) c-Jun NH2-terminal kinase (JNK) activation, causing pro-inflammatory activation of cells. When NFκB and MAPK JNK activation is impaired, fas-associated death domain (FADD) and procaspase 8 attach to complex 1, leading to caspase 8 activation. TRAIL-Rs and CD95 have a similar mechanism, where FADD and procaspase 8 attach directly to the receptors, activating caspase 8. In Alzheimer’s disease, astrocytes are believed to be an important source of TRAIL, promoting the extrinsic apoptosis pathway, especially in the late phase of the disease [14].

#### Necroptosis

Activation of death receptors can also induce necroptosis as shown in Fig. 2. Similarly as in apoptosis in necroptosis, complex I is formed, but in this case, it is unstable, leading to the formation of complex II. As mentioned in the previous paragraph, ubiquitination of RIPK1 is necessary for the stabilization of complex I. Still, by inactivation of CYLD, protein caspase 8 prevents RIPK1 de-ubiquitination thus promoting complex I stabilization and apoptosis [15]. However, when caspase 8 is insufficiently activated, CYLD remains active for and de-ubiquitinates RIPK1, destabilizing complex I. This results in the interaction of RIPK1 with FADD, TRADD, RIPK3, and caspase-8, leading to the formation of complex II, which allows RIPK1 to interact with RIPK3 through the receptor homology domain (RHD), promoting the formation of necrosome and the initiation of downstream signaling, resulting in necroptosis. Therefore, caspase 8 activation and RIPK3 ubiquitination seem to be crucial checkpoints in determining whether the cell undergoes apoptosis or necroptosis. In certain conditions, both apoptosis and necroptosis can be activated simultaneously [16].

#### Intrinsic Apoptosis Pathway

Caspase 8 activation during the extrinsic pathway can lead to the activation of the intrinsic pathway by cleaving a member of the Bcl2 (B-cell CLL/lymphoma 2) family protein, Bid (BH3 interacting-domain death agonis), into tBid (truncated BH3 interacting-domain death agonis). tBid is an important activator of the intrinsic apoptosis pathway [17]. Intrinsic pathway activation can also be induced by DNA damage, ischemia, oxidative stress, or lack of growth factors. This results in the activation of downstream proteins, including p53 and proteins from the Bcl2 family. The balance between pro-apoptotic (e.g., Bax) and antiapoptotic (e.g., Bcl2) Bcl-2 family member proteins is crucial in determining whether apoptosis occurs [18].

Pro-apoptotic Bcl2 family proteins have different mechanisms of action, dependent on what domains they contain. Proteins which contain only BH3 domain activate Bak (Bcl-2 homologous antagonist killer) and Bax (BCL2-associated X, apoptosis regulator), by the interaction of them with anti-apoptotic proteins, which otherwise blocks Bak and Bax. Still, some BH3-only proteins, like tBid, Bim (Bcl-2 Interacting Mediator of cell death) , and Puma (p53 upregulated modulator of apoptosis), may also directly activate Bak and Bax. On the other hand, anti-apoptotic Bcl2 family proteins negatively regulate apoptosis by forming complexes with pro-apoptotic Bcl2 family proteins [19]. The activity and expression of proteins from the Bcl-2 family are regulated, among others, by the p53 protein. The p53 through its DNA binding domain promotes transcription of pro-apoptotic Bcl family proteins (Bax) and inhibits transcription of anti-apoptotic Bcl-2 [20, 21]. Moreover, p53 may activate Bcl2 family proteins by direct protein–protein interactions. Still, p53 induces not only apoptosis but also cell cycle arrest and induces reparation of DNA damages.

The Bcl2 family interactions in general are crucial in determining the cell fate. If pro-apoptotic signaling dominates, Bak and Bax integrate into the mitochondrial membrane, which induces the generation of pores, which leads to the release of proapoptotic factors, especially cytochrome C, but also endonuclease G (endo G), apoptosis-inducing factor (AIF), SMAC/Diablo, and Omi serine proteases/HtrA2, from the mitochondria. Cytochrome C, which, together with the cytosolic proteins Apaf and procaspase-9, forms the apoptosome. To prevent cell death, a complex consisting of the inhibitors of apoptosis Bcl-2/Bcl-XL joins the apoptosome and blocks its functions. This complex is inactivated by tBid, Bad, and Bik proteins, causing the activation of procaspase-9. Caspase-9 is also able to convert Bid to its active form (tBid) by cleaving the peptide bond at the Asp59 position, making a positive feedback loop [22].

#### ER-Stress-Induced Apoptosis Pathway

**Fig. 3:**
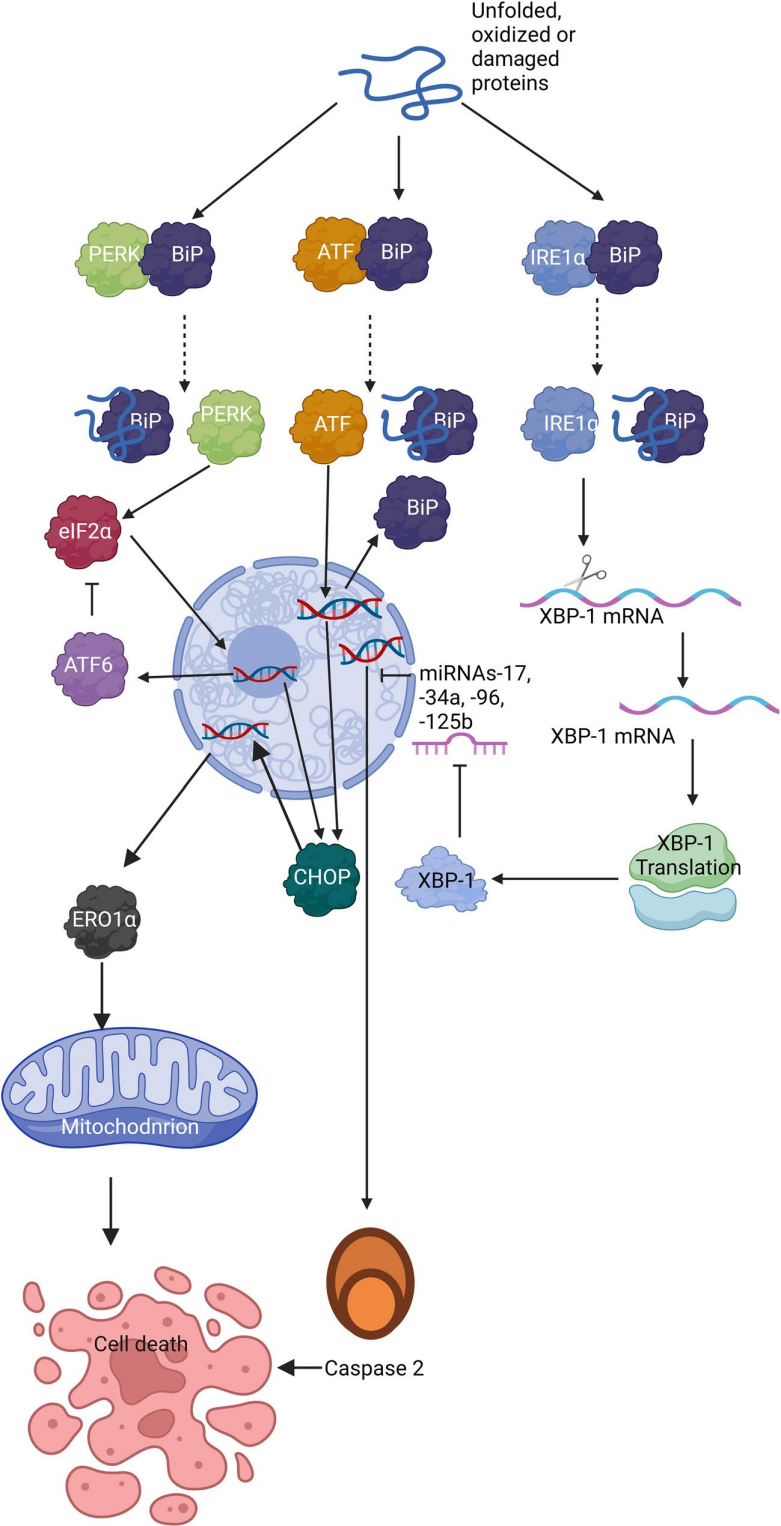
ER-stress-induced pathway

The third mechanism of apoptosis activation, ER stress-induced pathway, as the name suggests, is a response to ER stress, which occurs when unfolded, oxidized, or damaged proteins accumulate in the ER. This pathway involves the interaction of binding immunoglobulin protein (BiP) with these proteins, leading to the breakdown of BiP-PERK, BiP-ATF6, and BiP-IRE1α complexes, releasing PERK (R (PKR)-like endoplasmic reticulum kinase), ATF6 (activator transcribtion factor6), and IRE1α (inositol-requiring enzyme 1 α) [23]. Activation of PERK, ATF6, and IRE1α has two important biological consequences. It promotes the production of chaperones BiP and PDI and inhibits mRNA translation (Fig. 3). Therefore, transcription of new proteins is decreased which prevents the accumulation of further proteins, so protective mechanisms may counteract ER stress and restore physiological conditions.

Nevertheless, severe ER stress or ineffective protective mechanisms can lead to apoptosis and in this case, IRE1α can act as a kinase or endo-RNAse. It auto-phosphorylates itself after release, which leads to its full activation. Fully activated IRE1α cleaves the regulatory intron from the X-box binding protein-1 (XBP-1) mRNA, allowing XBP-1 to be translated. XBP-1 in turn upregulates caspase 2 transcription by degrading its repressors: miRNA-17, miRNA-34a, miRNA-96, and miRNA-125b [24].

Under ER stress, ATF6 undergoes limited proteolysis, which promotes its activation, as its cleaved N-terminal domain attaches to ER stress-response elements (ERSE-1) and ATF/cAMP response element (CRE). This promotes transcription of not only cytoprotective proteins like BiP chaperone and nuclear protein 1 (NUPR1), but also proapoptotic C/EBP homologous protein (CHOP). On the other hand, PERK inhibits the phosphorylation of eukaryotic translational initiation factor 2 (eIF2α), thus, decreasing its activity, which impairs total protein translation. On the other hand, eIF2α phosphorylation increases CHOP and ATF4 transcription. ATF4 increases GADD34 transcription, which dephosphorylates ATF6. Thus, its action leads to a negative loop. CHOP stimulates the transcription of protein phosphatase 1 regulatory subunit 15 (PPP1R15A/GADD34), TNF-related apoptosis-inducing ligand (TRAIL2), tribbles homolog 3 (TRB3), and endoplasmic reticulum disulfide oxidase 1α (Ero1α). Finally, Ero1α stimulates the transport of Ca2 + to mitochondria via the IP3R receptor, causing cell death [25, 26].

#### Pyroptosis

In contrast to apoptosis, pyroptosis is a type of cell death associated with inflammation. Typically, inflammasome formation begins when cytoplasmic pattern recognition receptors (PRR) are activated by pathogen-associated molecular patterns (PAMPs) or damage-associated molecular patterns (DAMPs). PAMPs are compounds characteristic of pathogens; they are commonly found in these organisms but are not produced in the human body, such as LPS, which is an essential element of the bacterial cell wall, so the presence of LPS is always associated with the appearance of bacteria [27]. On the other hand, DAMPs are released from dying cells due to trauma or an infection by a pathogen. After activation, PRR forms large complexes with pro-caspase 1 and apoptosis‐associated speck‐like protein containing a CARD (ASC). ASC contains two domains: a pyrin domain (PYD) and a caspase activation and recruitment domain (CARD). As the CARD domain plays a crucial role in caspase 1 recruitment in the case of some PRRs, which contain CARD, pro-caspase-1 can be directly recruited to PPR, and ASC recruitment in this case is not necessary. After inflammasome formation, caspase 1 undergoes hydrolyzation into 2 fragments and then forms a dimer. Activated caspase‐1 can cleave pyroptosis executor gasdemin D (GSDMD) proteins (Asp275 site) to free the N‐terminal (NT) domain. This domain makes nonselective pores with inner diameters of ~ 10–14 nm in the cell membrane leading to cell swelling and pyroptosis. Still, caspase 1 has also another important biological function. It cleaves the precursors of IL‐1β and IL‐18 to become mature IL‐1β and IL‐18. As these cytokines act pro-inflammatorily and, thanks to the permeabilization of cell membranes, are released from the cells, pyroptosis not only is activated by pro-inflammatory stimuli but also leads to a further increase in inflammation [28, 29].

## Alzheimer’s Disease

Alzheimer’s disease (AD) is a progressive and fatal neurodegenerative disorder manifested by cognitive and memory decline caused by irreversible neuronal loss, particularly in the cerebral cortex and hippocampus. It is associated with the deposition of insoluble forms of amyloid β (Aβ) in the form of plaques in extracellular spaces, as well as in the walls of blood vessels, and the aggregation of τ microtubule protein into neurofibrillary tangles in neurons [30]. Pathogenesis of AD begins with the accumulation of Aβ in the cortex or neurofibrillary tangles (NFTs), but these phenomena may precede the initial symptoms by many years. Later clinical manifestations can be divided into amnestic syndrome, which is associated with damage to the hippocampus, and focal symptoms associated with progressive aphasia and visual agnosia [31].

AD is the most common neurodegenerative disease, accounting for approximately two-thirds of dementia cases, with vascular and other neurodegenerative causes, such as Pick’s disease and diffuse dementia with Lewy bodies, accounting for the majority of the remaining cases [30]. It results in progressive impairment of activities of daily living and a variety of neuropsychiatric and behavioral symptoms. Characteristic clinical features include progressive impairment of memory, judgment, decision-making, orientation to the physical environment, and speaking. Most cases of AD are not dominantly inherited but many people with AD have a complex genetic association [32]. It is estimated that in 2020, there were approximately 50 million people worldwide with Alzheimer’s disease. Older people with low levels of education, vascular disease, and exposure to environmental factors are particularly vulnerable to the disease. The aging of the population is part of a demographic trend—as the “baby boomer” generation in developed countries reaches old age, the problem of AD and dementia more broadly will increase. Globally, the number of elderly people (over 65 years of age) will increase dramatically; the number of elderly people is projected to rise to an overwhelming number of 973 million by 2030 (up from 420 million in 2000) [33]. 

Genetic risk factors include rare, dominantly inherited mutations in APP (encoding amyloid precursor protein on chromosome 21q21.3), PSEN1 (encoding presenilin 1 on chromosome 14q24.3), and PSEN2 (encoding presenilin 2 on chromosome 1q31-q42) and in more common but incompletely penetrant genetic variants such as APOE [31, 34]. Head trauma, level of education, and other risk and protective factors identified in epidemiological studies may influence the likelihood of developing AD by affecting brain reserve—the brain’s ability to withstand the increasing burden of amyloid without exhibiting dysfunction and cognitive impairment [35, 36].

Other risk factors for all forms of dementia also include smoking, depression, low physical activity, social isolation, diabetes, hypertension, and air pollution; however, it is difficult to distinguish the cause and effect of dementia for some of these (e.g., depression) [37, 38]. However, studies of cerebrospinal fluid biomarkers or PET scans show that vascular risk factors influence the development of dementia and the form of AD, but not the cause [39–41].

## Role of Caspases in the Pathophysiology and Pathogenesis of AD

**Fig. 4:**
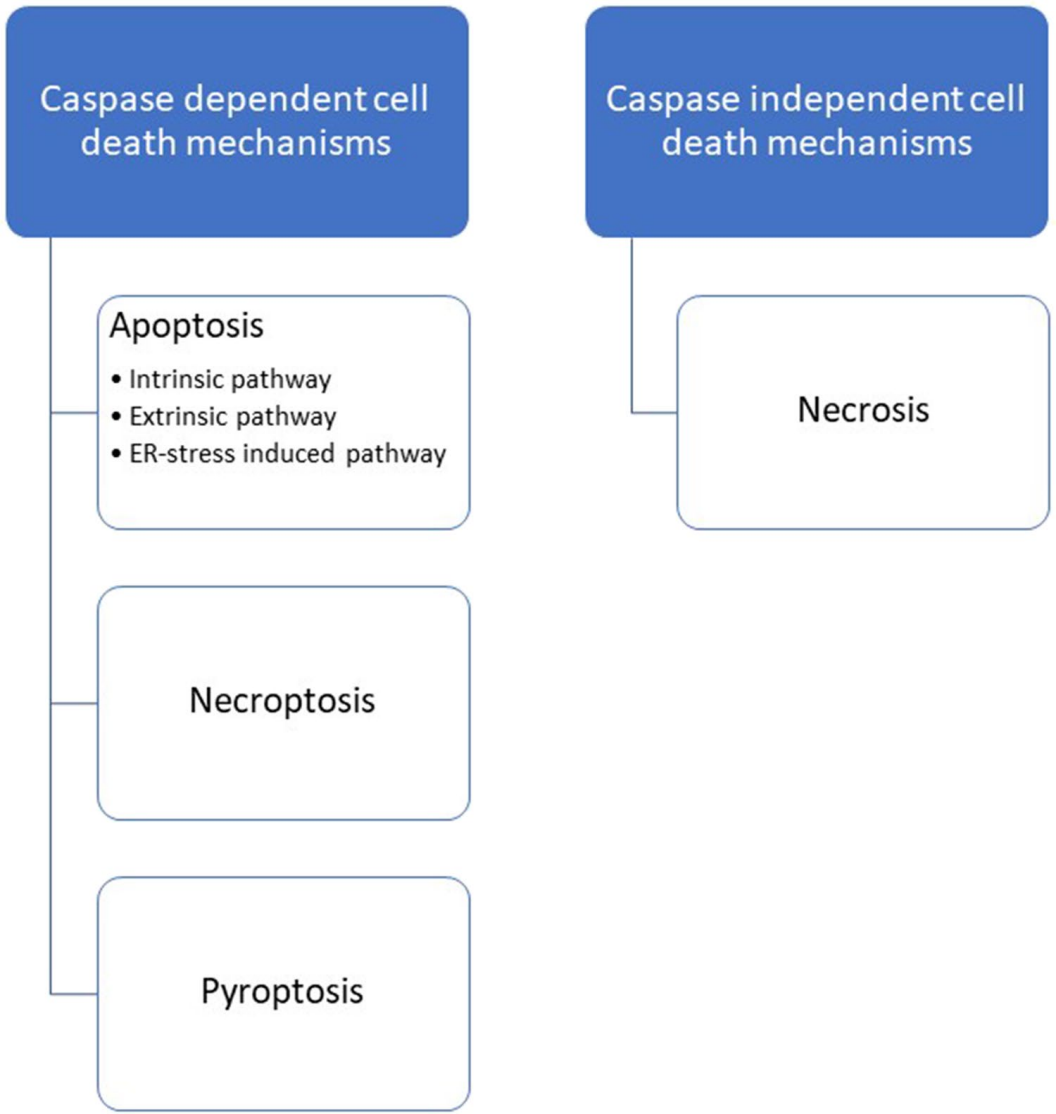
Different mechanisms of cell death observed in AD

Several mechanisms have been described to explain the pathology of AD. Some of the proposed mechanisms of AD include the Tau hypothesis, the amyloid cascade hypothesis, excitotoxicity, the cholinergic hypothesis, and others, such as the graph theory [42]. However, all these theories have one thing in common. According to each of them, at some point in the development of Alzheimer’s disease, there is an increased death of nerve cells, for which various mechanisms may be responsible as pointed out in Fig. 4.

As neurons are cells with very limited proliferation, their increased death can easily cause a decrease in their number; therefore, increased apoptosis of neurons, which is observed in the case of AD, has a very large impact on the functioning of the brain as a whole. Nevertheless, despite the fact that apoptosis has a negative impact on the functioning of the AD brain, in general, it is a physiological, precisely regulated mechanism of cell death evolutionarily designed for the removal of unnecessary or damaged cells. Therefore, in the human body, this process is repeated all the time in different cell populations. Moreover, apoptosis does not increase inflammation, as cell residues are removed and phagocytosed by the macrophages [43]. Additionally, it is also necessary for body development, immunoregulation, and cell protection against DNA damage, which otherwise could result in tumor development. Still, although apoptosis has a rather positive impact on the functioning of the human body, during some pathological conditions, apoptosis can be excessively increased, which, in turn, leads to the degeneration of organs and tissues. During pathological conditions, other mechanisms of cell death can be activated. Therefore, as both insufficient and excessive apoptosis can have catastrophic consequences for the organism, apoptosis needs to be precisely regulated, which involves the action of apoptosis inhibitors and activators. Nevertheless, the aforementioned pathways can occur simultaneously, and one of them can activate others [12]. Therefore, even though all of the aforementioned pathways are activated during AD, and increased activation of caspases 2, 8, or 9 is observed, it is very difficult to determine which pathway is the first one to be activated or which is the most important one.

Expression of activated caspases 3 and 8 is observed in AD brains, confirming the role of the extrinsic pathway in the apoptosis of neurons in AD, which can also be linked with Aβ deposits. An increased level of activated caspase 9 is observed in the hippocampus of Alzheimer’s disease patients. Moreover, there is also a correlation between activated caspases 8, 9, and 3 in the same neurons, suggesting that the intrinsic pathway is activated by the extrinsic pathway in the case of AD. On the other hand, the co-localization of DNA damage and caspase 9 in AD suggests that DNA damage is the leading factor causing caspase 9 activation in AD [18]. This also corresponds to the upregulation of p53, which is a DNA damage sensor and apoptosis activator in AD [44]. Unfolded protein response is observed in AD, as the increased level of neurons positively stained for Ero1α, IRE1α, and PERK is increased in AD [45]. Especially, IRE1 may be involved in the regulation of Aβ-induced cell death as a positive correlation between IRE1 activity and the progression of AD is found in AD patients, as well as increased caspase 2 activation. This strongly suggests that activation of caspase 2 in AD is mediated by IRE1 [46].

Moreover, all of these pathways lead to the same common molecular features, which are also observed in AD. Activated initiator caspases activate executioner caspases 3, 6, and 7, which are responsible for the degradation of intracellular proteins. Among them, caspase 3 is believed to be the most important, and its activation is one of the markers of apoptosis; therefore, increased caspase 3 activity in AD neurons is believed to be a hallmark of increased apoptosis of these cells [47, 48]. Among the different proteins that are proteolyzed by caspase 3, DFF45/ICAD, an inhibitor of DFF45/CAD, seems to be especially important. Degradation of DFF45/ICAD leads to the activation of DFF45/CAD, which is responsible for the fragmentation of DNA into 180 bp or larger fragments that are multiples of 180, such as 360 bp or 540 bp. This is another important molecular feature of apoptosis, as in other mechanisms of cell death, DNA is fragmented in a random way. Therefore, some methods of assessing apoptosis, like TUNEL (Terminal deoxynucleotidyl transferase dUTP nick end labelin) methods, are based on the detection of these fragments, and TUNEL assessment also confirmed increased apoptosis of AD neurons [49]. Another important marker of apoptosis is the externalization of phosphatidylserine, a molecule that normally is present only in the inner layer of the cell membrane. Nevertheless, during apoptosis, phosphatidylserine moves from the inner to the outer layer of the cell membrane, being a signal for macrophages and similar cells to phagocytose cell fragments after apoptosis [50], and the loss of phospholipid asymmetry was also observed in AD brains using immunostaining [51].

Additionally, cells isolated from AD patients are more prone to undergo pyroptosis, which additionally suggests dysregulation of negative regulators of pyroptosis or overactivation of positive pyroptosis regulators in AD. Additionally, upregulation of NLRP1 and NLRP3 is observed in different cell populations in AD patients as well as in a mouse model of AD, which suggests that the upregulation of these proteins is the reason for the increased pyroptosis in AD [52]. Nevertheless, there is not only evidence that pyroptosis is overactivated in AD, but also that it plays an important role in AD pathophysiology. Research on a mouse model of AD demonstrates that the knockdown of caspase 1 not only leads to decreased pyroptosis, as observed by lower expressions of NLRP3, caspase-1, and GSDMD, but also leads to an improvement in the cognitive functions of animals.

Still, despite the fact that increased neuronal death and increased caspase activation are widely known features of AD, the exact molecular mechanisms that lead to this are not completely understood. One of the reasons may be Aβ or Tau actions, as these proteins are known for their pro-apoptotic actions. The answer to these questions seems to be crucial in understanding the role of caspases in the pathophysiology of AD and better understanding this disease. Therefore, the role of different apoptotic pathways in AD and how they contribute to the current knowledge of this disease is more detailed in the description provided below.

### Cross-talk Between Amyloid β and Caspases

The amyloid cascade theory initially hypothesized that the accumulation and deposition of A peptide and β plaques in the central nervous system have a strong correlation with dementia and play an important role in the pathogenesis of AD. Aβ is a 40- or 42-amino-acid peptide derivative of amyloid precursor protein (APP), a transmembrane protein obtained by sequentially cutting β-secretase (BACE1) and γ-secretase. τ is a microtubule-associated protein that enhances the polymerization and stabilization of microtubules in the cell cytoskeleton [32, 53].

However, it has been found that amyloid plaques also deposit in healthy brains with age and that A-peptide accumulation and subsequent deposition are not associated with neuronal loss or cognitive decline [54]. The hypothesis has evolved to assume that Aβ is not masked in the plaques but drives the disease [34]. AD brain cortical plaques are mainly composed of Aβ protein. Aβ is produced by processing its parent protein, APP. The specific physiological roles of APP are not entirely clear, but it is generally thought to contribute to normal neuronal function and perhaps brain development. The amyloid hypothesis suggests that degradation of APP-derived Aβ by secretase decreases with age or pathological conditions, leading to the accumulation of Aβ peptides (Aβ40 and Aβ42). An increase in the Aβ42/Aβ40 ratio induces the formation of Aβ amyloid fibrils, resulting in neurotoxicity and the induction of τ pathology, which ultimately leads to neuronal cell death and neurodegeneration [55].

**Fig. 5:**
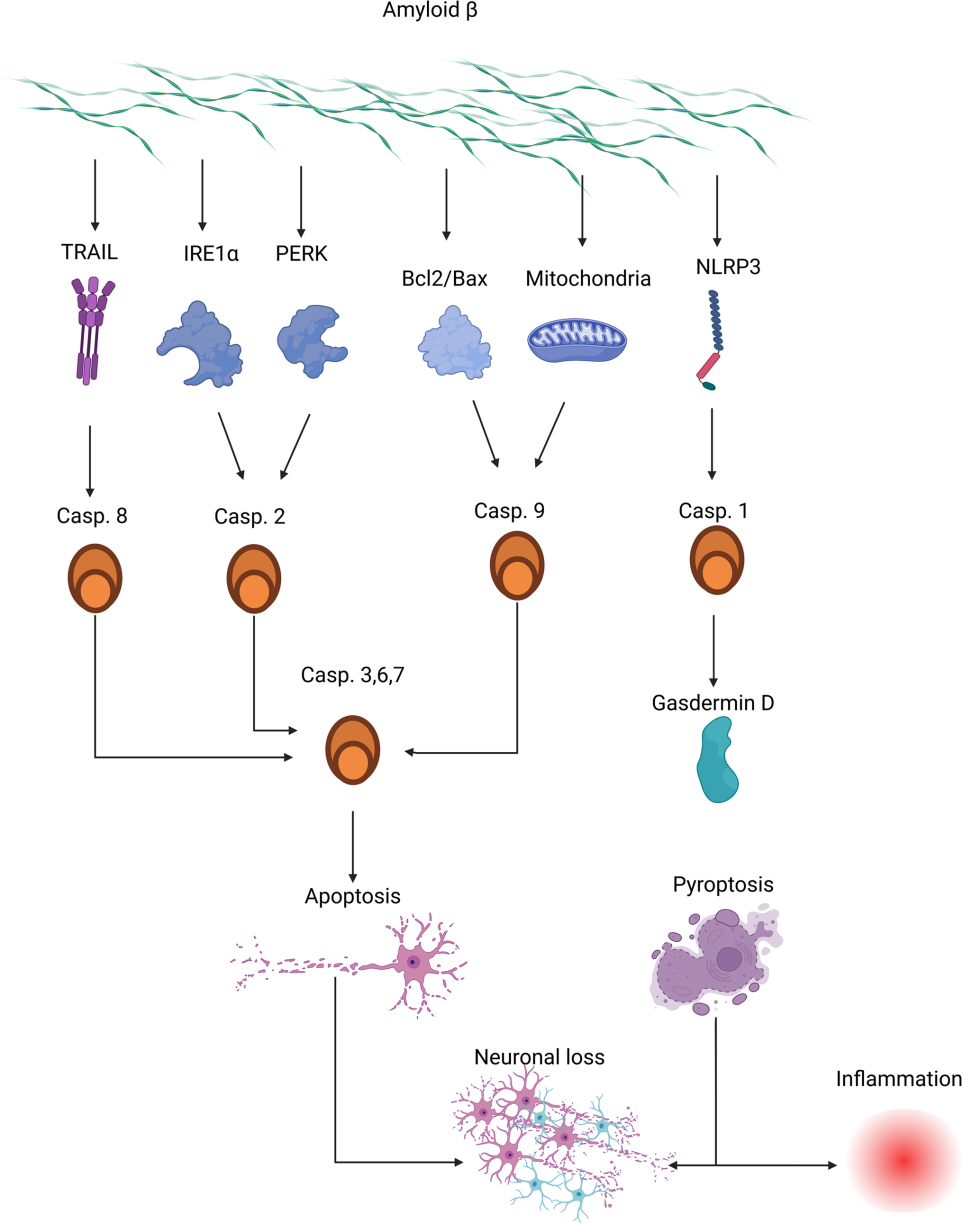
Role of Aβ in activation of caspases in AD

Processing of APP occurs through a series of bursts that involve initial degradation by enzymes with α-secretase activity and β-secretase BACE1. The α-secretase-induced excision leads to the formation and release of an amino-terminal peptide called APPs α, which is present in reduced amounts in AD patients and is associated with neuroprotective functions [56]. BACE1 expression may be modulated by frequently observed situations in neurodegenerative diseases and aging [34, 53]. Nevertheless, APP can be cleaved by caspases, which correlate with synaptic loss and cognitive dysfunction. Caspase 3 is believed to be responsible for that process, and its inhibition decreases APP cleavage [57]. To check if increased APP cleavage by caspases is a correlation or cause-and-effect action, researchers generated an APP D664A KI mouse line by homologous recombination with an Asp (D) to Ala (A) substitution. As caspases act selectively on Asp, replacing this amino acid with another makes the protein insensitive to caspases. Hippocampal organotypic slice cultures (OTSCs) were obtained from these mice and used for in vitro examinations when they were proved to be resistant to Aβ-induced cytotoxicity [58]. Nevertheless, a similar observation is in the case of caspase 8, in which case deletion of caspase 8 and RIPK3 leads to decreased Aβ deposition in brains when deletion of only RIPK3 does not cause such an effect [59]. This shows that caspases play a role in AD that is contrary to the classical understanding of their role in human physiology. Most people consider caspases to be proteins whose role is limited to the later stages of various cell deaths [60, 61]. However, in the case of Alzheimer’s disease, their role seems to be much wider. In this case, caspases play a very important role in the early stages of the disease as their action is necessary for the development of Aβ plaques, which are the initial stages of the development of Alzheimer’s disease, leading to further consequences. However, it is debatable which specific caspases are the most important in this process, as AD is accompanied by an increase in the activity of caspases 2, 3, 8, and 9 (Fig. 5). Still, in vitro examinations show that the leading role in that case is played by caspases 3 and 8. Therefore, activation of the extrinsic pathway, which leads to the activation of both caspases 3 and 8, seems to be crucial. It is important to remember that activation of caspase 3 is a downstream event to activation of caspase 8, so observed changes in Aβ metabolism [59] may not be an effect of the direct action of caspase 8, but the impact of caspase 8 on caspase 3. Therefore, the exact role of caspase 8 is not known and should be further investigated in the future.

Nevertheless, these observations indicate an important role of neuroinflammation in AD pathophysiology. AD is accompanied by neuroinflammation and higher levels of pro-inflammatory mediators, especially TNFα, are observed in this disease. TNFα activates death receptors, which in turn induce the extrinsic apoptotic pathway and activate caspases 3 and 8. This leads to the generation of Aβ, which is also a pro-inflammatory factor, creating a potential positive feedback loop. Inflammation induces caspase 8 activation, which promotes Aβ deposition, further triggering caspase 8 activation. There is also a possibility that Aβ interacts more directly with death receptors [62], suggesting that the impact of Aβ on AD is multi-targeted. The translocation of amyloid β to the brain across the blood–brain barrier via receptors, mainly receptors for advanced glycation end products (RAGEs), is an important issue [63]. Interactions of RAGE with amyloid β cause inflammatory reactions at the endothelial level and apoptosis of endothelial cells, which have a significant impact on the neurovascular changes observed in AD [53, 64].

Aβ can also induce apoptosis not only in endothelial cells but also in neurons. Caspase 8-dependent apoptosis has been observed in PC12, a rat neuronal cell line, after Aβ treatment. Knockdown experiments using siRNA targeting caspase 8 showed a significant decrease in apoptosis, as well as caspase 9 and 3 activation, in Aβ-treated cells compared to non-transfected Aβ-treated cells [65]. However, despite the evidence pointing to increased necroptosis in AD, caspase 8 silencing had no effect on Aβ-induced necroptosis [66, 67]. This suggests that increased caspase 8 activation observed in AD promotes cell death rather than protecting cells against necroptosis [65]. The exact mechanism of Aβ-dependent caspase 8 activation is not completely understood. Some researchers suggest that Aβ may act via death receptors, as it induces the expression of TRAIL in cells. Co-localization of TRAILs 1 and 2 with Aβ has been observed, and western blot analyses have confirmed the binding of Aβ to TRAILs [62]. Knockdown of TRAILs also protects cells against Aβ-induced apoptosis, further suggesting the necessity of TRAIL activation for caspase 8 activation in AD. Overall, these studies suggest that the activation of death receptors, particularly TRAIL, by Aβ leads to the activation of the extrinsic pathway and subsequently activates caspase 8 and 3, leading to neuronal loss.

Aβ also induces caspase 9 activation. Aβ alters the balance between Bcl2 and Bax proteins in favor of the pro-apoptotic Bax, which leads to caspase 9 activation. Studies using Bax^−/−^ mice have shown that these animals are partially resistant to Aβ-induced apoptosis, confirming the role of modulating the Bcl2/Bax balance in caspase activation by Aβ [68]. The protein PUMA is also believed to be important in the regulation of neuronal apoptosis [69]. Aβ administration induces upregulation of PUMA, and PUMA silencing protects cells against Aβ-induced apoptosis [70]. However, some researchers do not confirm caspase 9 activation after Aβ treatment [71]. They suggest that Aβ, by binding to procaspase 9, may inhibit apoptosome formation and caspase 9 activation [72]. Unfolded protein response may also be activated by Aβ or Tau, as observed in different human cell populations [73, 74]. In particular, the PERK-eIF2α pathway is activated in Aβ-treated human cholinergic neuroblastoma cells, and PERK knockdown protects these cells against the pro-apoptotic effects of Aβ [73]. In addition to apoptosis, it is well known that Aβ promotes the release of IL-1 from cells, likely through caspase 1 action and pyroptosis [75–77]. This process is mediated by the activation of NLRP3, one of the pattern recognition receptors that can be activated by Aβ. Genetic deletion of caspase 1, NLRP3, or ASC inhibits IL-1 release, suggesting the crucial role of inflammasome generation and caspase 1 activation in IL-1 release after Aβ administration. Caspase 8 also plays a role in this process, as its deletion significantly reduces the expression of NLRP3 after Aβ administration [59, 78].

Therefore, we can observe an intriguing cross-talk between Alzheimer’s disease, Aβ, and caspases. Caspases induce Aβ cleavage and deposition, and Aβ can induce the activation of different caspases in AD. However, like most complex biological interactions, this is not completely understood. It is known that Aβ induces caspase 8 activation, which triggers downstream events such as caspase 3 activation. Less is known about the intrinsic pathway, as research is inconclusive, with some studies suggesting that caspase 9 and the intrinsic pathway are activated by Aβ, while others do not. There is even more confusion about how caspase 9 activation occurs, as there are three potential pathways involved. The first is the effect of Aβ on proteins from the Bcl2 family, especially Bcl2 itself and Bax. The second is the effect of Aβ on mitochondrial proteins, and the third is the activation of caspase 9 through the activated Bid protein, which is the result of caspase 8 action. Unfolded protein response may also play a role in Aβ-induced caspase activation, although its influence has not been demonstrated conclusively. Particularly intriguing is the situation with pyroptosis, as different caspases are involved in this process, and Aβ can influence it in various ways. Aβ increases the expression of death receptors by inducing caspase 8 activation. Additionally, by interacting with NLRP3, Aβ induces caspase 1 activation and pyroptosis. This leads to inflammation, which further activates caspase 8, ultimately leading to apoptosis and Aβ deposition. Taken together, these findings suggest the crucial role of caspase 8 activation in the context of the Aβ hypothesis.

The amyloid cascade hypothesis is widely accepted, but research supports the view that amyloid β is not the sole contributor to AD pathogenesis.

### Cross-talk Between τ Protein and Caspases

The τ hypothesis is, in part, an extension of the amyloid cascade hypothesis. In vitro experiments using neuronal cell lines, primary hippocampal and cortical neurons, and organotypic cultures of the hippocampus have shown that amyloid β induces changes in τ protein [79, 80]. Changes in τ protein and amyloid β oligomers are listed as the most important factors responsible for neuronal dysfunction in AD pathogenesis [81]. The description of τ pathology correlates better with cognitive impairment than Aβ damage, but there is no uniform theory to explain its important role [53].

The τ protein is a highly soluble protein that binds to microtubules and promotes functions such as structural changes, axonal transport, and neuronal growth. A large accumulation of τ protein in the hippocampus characterizes a subtype of AD called the “limbic-predominant type,” while “hippocampal sparing” refers to a milder accumulation of τ in the hippocampus compared to the expected pathology [82]. In the τ hypothesis, it is assumed that hyperphosphorylated τ protein arises as a secondary pathogenic event, leading to its displacement from microtubules and subsequent aggregation into paired helical filaments (PHFs) and NFTs, resulting in neurodegeneration prior to plaque A formation. The Apo epsilon 4 (APOE4) allele of the apolipoprotein (APO) gene, encoding a protein involved in cholesterol metabolism and lipid transport, is thought to play an important role [53, 83, 84].

According to this theory, caspases also play a role in the development of AD. In vitro studies have shown that caspases 2, 3, 7, and 8 can cleave Tau into two fragments, and caspase-3-cleaved Tau aggregates more easily than full-length Tau, thereby increasing aggregation of full-length Tau [85]. Moreover, cleaved Tau has been found to be toxic to neurons [86]. Immunostaining of AD brains confirms the role of caspases and caspase-cleaved Tau in the pathogenesis of AD, as the caspase-cleaved carboxy-terminus of Tau is present in large amounts in AD patient brains [85]. Caspase 3, cleaved Tau, and fibrillary Tau are colocalized in AD brains, and activated caspases are also present in most cells with NFTs [87, 88]. These observations lead to the conclusion that caspase-dependent Tau cleavage contributes to the development of cognitive dysfunction and AD [89]. Animal research has partially confirmed this, as increased caspase activity correlates with Tau expression and cognitive dysfunction. Similar conclusions can be drawn for caspase 2, where the preparation of Tau resistant to caspases or caspase 2 deficiency acts cytoprotectively in vitro and improves cognitive functions in mouse models [90, 91].

However, the role of Tau in caspase activation remains unclear. On one hand, Tau can activate caspase 2 by inducing an unfolded protein response. On the other hand, some researchers suggest that Tau can inhibit caspase 9 activation. Both of these processes may be due to the same reason. Unfolded protein response can induce p53 activation. However, the situation is more complex, as recent research suggests that despite upregulation in AD, the action of p53 is impaired due to aggregation with Tau, preventing its translocation into the nucleus [92]. Therefore, the positive impact of p53 on DNA damage repair may not be induced. On the other hand, p53 activation can also occur during oxidative stress or due to mitochondrial failure.

### Contribution of Caspases and ROS to the Mitochondrial Cascade Hypothesis

**Fig. 6:**
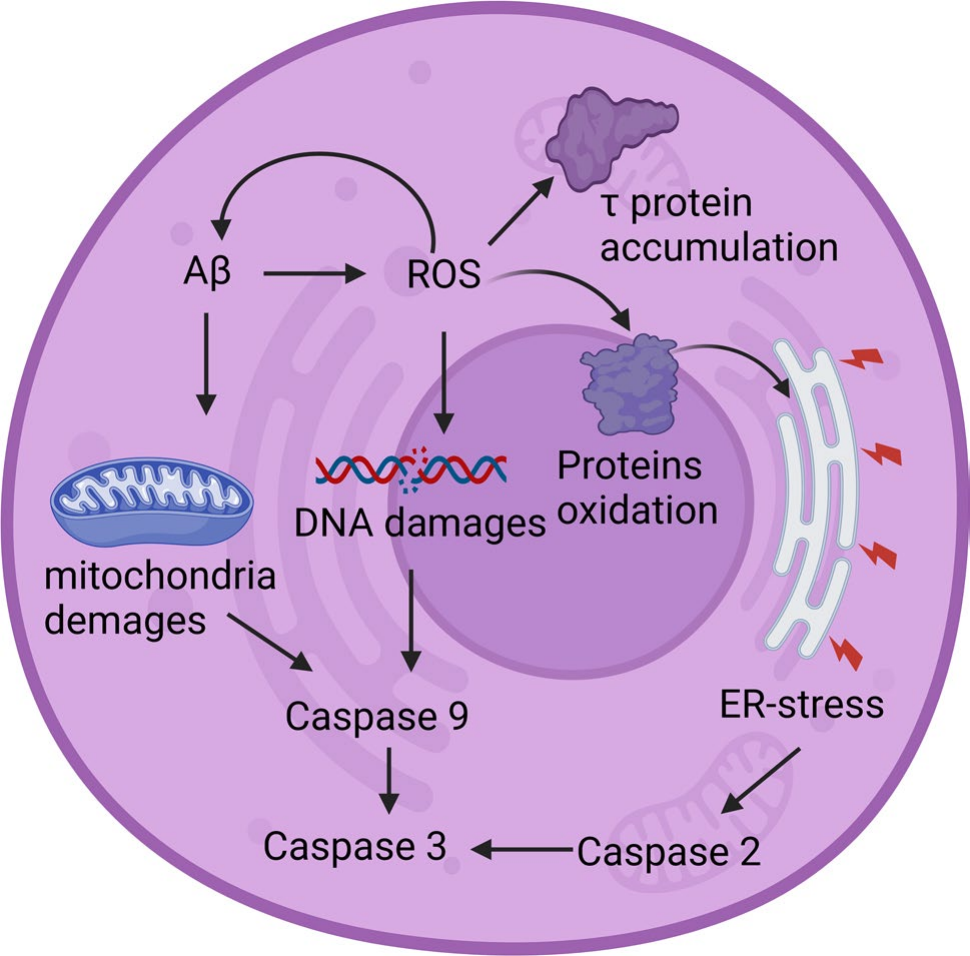
Role of mitochondria and ROS in activation of caspases in AD

The mitochondrial cascade hypothesis posits that similar physiological mechanisms underlie AD and brain aging and that mitochondrial dysfunction in AD is not merely a consequence of neurodegeneration (Fig. 6). It further suggests that mitochondrial dysfunction drives amyloidosis, Tau phosphorylation, and re-entry into the cell cycle. This hypothesis extends the free radical theory of aging by incorporating information on the role of somatic mitochondrial DNA (mtDNA) damage. According to this theory, various mechanisms, including oxidative stress and proteasome dysfunction, contribute to mitochondrial dysfunction in neurodegenerative diseases such as AD [93].

AD patients exhibit lower cytochrome oxidase activity in platelet samples containing mtDNA, and AD hybrids with reduced activity produce excessive amounts of Aβ42 and Aβ40. There is a connection between mitochondrial function and amyloidosis, as mitochondrial electron transport chain (ETC) dysfunction increases free radical production [94]. Additionally, APP, Aβ, and the entire γ-secretase complex are found in mitochondria or mitochondrial membranes [95].

Neuronal apoptosis in neurodegenerative diseases like AD is known to be linked to oxidative stress (OS) [96]. Based on this hypothesis, the production of reactive oxygen species (ROS) is increased within mitochondria under certain stress conditions, including aging. In the absence of an effective antioxidant system, this augmented ROS production increases the likelihood of developing AD [84]. ROS interact with biological molecules and damage cells, as evidenced by the presence of free radicals in AD patients [97, 98].

Abnormal accumulation of amyloid β has been shown to promote ROS formation through the activation of NMDA receptors, and ROS can increase amyloid β production, aggregation, τ phosphorylation, and polymerization [99]. Increased ROS levels constitute a self-perpetuating process that contributes to the development of AD and ultimately leads to cell death through caspase activation and apoptosis [53, 100, 101].

As mentioned above, mitochondrial failure and ROS generation play important roles in the induction of apoptosis, particularly through the intrinsic pathway (Fig. 6). Mitochondrial damage can result in the release of pro-apoptotic factors, and due to their high energy demands, neurons are particularly sensitive to mitochondrial damage [102]. Moreover, Aβ can interact with mitochondrial proteins like cytochrome C oxidase, promoting mitochondrial damage and leading to the release of different factors from mitochondria, ultimately resulting in apoptosis [103, 104]. Furthermore, ROS can interact with cellular proteins, leading to endoplasmic reticulum (ER) stress.

**Fig. 7:**
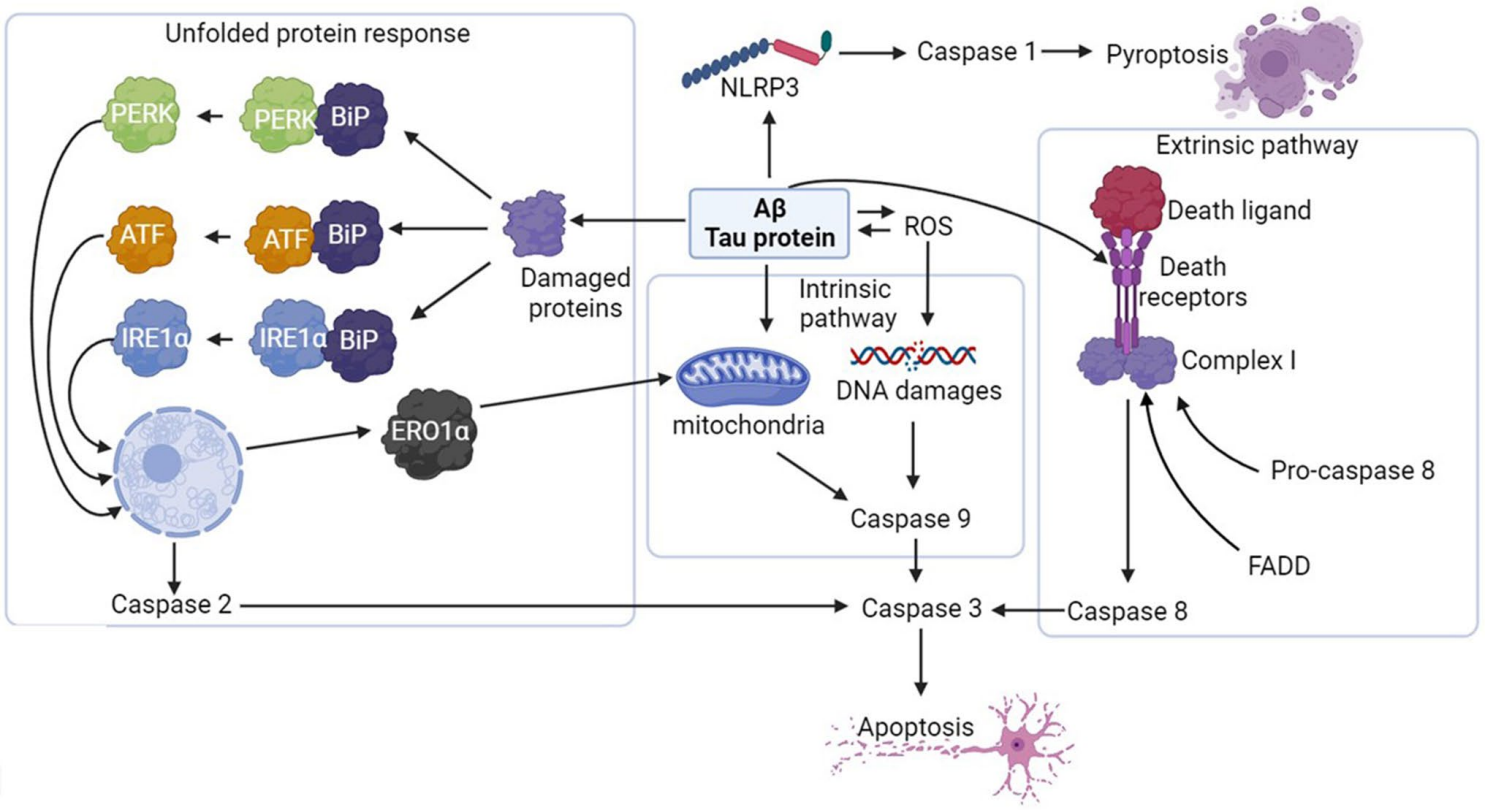
Different ways of caspase activation in AD

Therefore, caspase action may have different impacts on AD pathophysiology. The first is directly related to their role in the apoptosis process, which leads to neuronal loss and cognitive impairment (Fig. 7). In AD, increased activity of caspases related to each main apoptosis pathway is observed, and studies involving the deactivation of caspases indicate that the increase in their activity and apoptosis not only accompany but also significantly contribute to the clinical features of AD. However, it is still unknown whether one apoptosis pathway is the leading one or not. Some data suggest the activation of the intrinsic pathway and caspase 9 as a downstream effect of caspase 8, while others suggest that it can occur independently. This issue is particularly important in the context of various theories on the origin of Alzheimer’s disease. The intrinsic pathway is a response to oxidative stress, DNA damage, or mitochondrial failure, and its activation serves as a significant marker of mitochondrial damage. If caspase 9 activation were solely an effect of caspase 8 and not oxidative stress, it would suggest that mitochondria and ROS have a negligible effect on neurons in Alzheimer’s disease, thereby implying that the mitochondrial theory of Alzheimer’s development is incorrect. However, even if caspase 9 is activated completely independent of caspase 8, it would not allow for unambiguous conclusions regarding the leading role of mitochondria, as it could also be the effect of Aβ on mitochondria and proteins regulating the intrinsic pathway. Additionally, the role of the ER stress-induced pathway should not be overlooked. Its activation, while confirmed, is also challenging to pinpoint precisely in terms of the role it plays and which of the main theories it supports. On one hand, it is evident that Aβ accumulation can activate the unfolded protein response (UPR) and caspase 2. On the other hand, this pathway may be activated by oxidatively damaged proteins, so mitochondrial damage and ROS release would also activate this pathway.

AD is accompanied not only by apoptosis but also by pyroptosis and necroptosis, which are also regulated by caspases (1 and 8, respectively), leading not only to neuronal loss but also to inflammation. Furthermore, during neuroinflammation, pro-inflammatory molecules are produced, including TNFα, which, through TNFR, increases the activation of caspases 1 and 8. This can lead to a positive feedback loop in which pyroptosis and necroptosis activate themselves as well as the extrinsic pathway of apoptosis, ultimately resulting in neuronal loss.

These processes can be induced by Aβ and Tau protein, but on the other hand, Aβ and Tau not only activate caspases but also are processed by them into more toxic derivatives that have a greater tendency to form deposits in cells, representing the second mechanism by which caspases interact with AD pathophysiology. In this case, another positive feedback loop can be observed, as caspase activation leads to increased Aβ and Tau deposition, which, in turn, increases caspase activation, resulting in the generation of more pathological Aβ and Tau. Thus, even a slight trigger factor, such as pro-inflammatory cytokines or ROS, which leads to mild caspase activation, may ultimately result in significant caspase activation and massive neuronal loss. Therefore, modulating caspase action may be a potential therapeutic approach against AD, and clinical trials of caspase inhibitors as potential drugs for Alzheimer’s disease have been initiated.

## Preclinical and Clinical Studies on Caspase Inhibitors as Potential Pedication for Alzheimer’s Disease

The drug discovery pipeline involves numerous steps aimed at testing the efficacy and safety of a drug candidate in various conditions. This process consists of two main stages: preclinical and clinical trials. The primary goal of preclinical studies is to evaluate the safety, efficacy, pharmacokinetics, and pharmacodynamics of a drug candidate in animal models before proceeding to human trials. This stage requires a wide range of experiments, including in vitro assays to assess the molecule’s mechanism of action, in vivo studies using animal models to evaluate the safety of the drug, its efficacy, and pharmacokinetics in a more complex living organism, as well as separate toxicity testing to collect data on the influence of the compound on various organs, tissues, and reproduction/development. If the drug candidate shows promising results in preclinical studies, it may progress to human clinical trials, where more information on its safety and efficacy is obtained in controlled settings with human subjects [105, 106]**.**

### Preclinical Studies on Caspase Inhibitors

In recent years, there has been a growing interest in compounds with neuroprotective properties. Particularly, molecules that can mitigate neuronal damage resulting from caspase-dependent cell death and inflammation are considered a promising strategy for addressing neurodegenerative disorders. The first caspase inhibitor, known as the cytokine response modifier (CrmA), was discovered as a product of the cowpox virus [107–110]. This discovery paved the way for a wide group of naturally derived caspase inhibitors, extensively described in the literature. A reader interested in learning more details about this group of compounds can refer to a comprehensive review article by Dhani et al. published in 2021 [110].

Thus, no surprise that a wide group of herbal ingredients was tested for their neuroprotective properties. For example, a study by Thenmozhi et al. examined the potential of hesperidin, a flavonoid found in citrus fruits, to counteract AlCl_3_-induced apoptosis in Wistar rats. Co-administration of hesperidin with AlCl_3_ significantly reduced the levels of cytosolic cytochrome c, caspase-3, caspase-8, and caspase-9 in animals with neuroinflammation caused by prolonged AlCl_3_ treatment [111]. Wen et al. investigated a group of compounds, including trans-4-hydroxystilbene, trans-3,5,4′-trihydroxy-stilbene (resveratrol), and trans-3′,4′,3,5-tetrahydroxy-stilbene (piceatannol), for their neuroprotective and antioxidant activity against Aβ-induced neurotoxicity in rat primary cortex neurons. The results confirmed the neuroprotective ability of these compounds and showed that, among others, they suppressed the activation of caspase-9 and caspase-3 [112]. Later, a study by Lei et al. assessed the involvement of pinoresinol diglucoside in neuroinflammation and neuronal apoptosis in mouse models of Alzheimer’s disease. Western blot assays indicated that this molecule increases the ratio of Bcl-2/Bax and downregulated cytochrome c and cleaved caspase-3 expressions, thereby inhibiting neuronal apoptosis [113]. Unfortunately, these effects were not attributed to a direct interaction with any subtype of caspases but rather to an indirect stimulation of alternative pathways. Interestingly, a study published in 2020 by Huang et al. evaluated the protective effect of vitamin K2 against amyloid-β-induced neurotoxicity. Experiments revealed that this chemical reduces the formation of reactive oxygen species and inhibits the caspase-3-mediated apoptosis induced by amyloid-β [114]. More recently, Moreno et al. summarized the result of their search for novel caspase-1 inhibitors isolated from Brazilian marine invertebrates belonging to two different phyla. The authors managed to identify one extract, derived from *Chiropsalmus quadrumanus*, which possessed the activity of caspase-1 inhibition in enzymatic assays. Subsequent purification efforts led to the conclusion that trigonelline, an alkaloid commonly found in plants, could be the compound responsible for caspase inhibition [115].

Similarly, as in the case of other problems addressed by rational drug discovery, there has been a noticeable shift from nature-derived products to synthetic molecules. Therefore, there is now a diverse library of small molecules and peptide inhibitors capable of interacting with various subtypes of caspases. Flores et al. studied VX-765, a compound that crosses the blood–brain barrier, and has been shown to reverse episodic and spatial memory impairment in animal models of Alzheimer’s disease by inhibiting caspase-1. However, the positive effects on cognition were reversed in animals excluded from the dosing regimen for more than a month [116]. In a study published by Zhou et al., the influence of methylene blue on the development of age-dependent cognitive impairment, neurodegeneration, and neuroinflammation was evaluated in animal models of AD. This therapy rescued caspase-6-induced episodic and spatial memory deficits, which were measured in behavioral tests of novel object recognition and Barnes maze. Methylene blue exhibited this effect by inhibiting caspase-6 and caspase-6-mediated neurodegeneration and neuroinflammation, which could potentially benefit patients suffering from cognitive decline in AD, if translatable to human studies [117]. Another manuscript, targeting the same subtype of caspase was published by Tubeleviciute-Aydin et al. In this paper, the authors aimed to develop novel allosteric inhibitors against active casp-6 and applied virtual screening against the putative allosteric pocket of the target to narrow down the initial library of 57,700 compounds. Computational analysis was enriched by in vitro data and led to the identification of novel compounds characterized by IC_50_ and K_i_ values ranging from 2 to 13 µM [118]. Finally, Bresinsky et al. published a study where they explored the binding pocket of caspase-2 and caspase-3 using computational tools, in order to determine the binding interactions crucial for the development of selective inhibitors. They utilized this information to design and synthesize 35 novel inhibitors based on the structure of the canonical inhibitor AcVDVAD-CHO and the Tau cleavage sequence YKPVD, which were further evaluated for their inhibitory activity at casp-2 and casp-3. As expected, some of these peptides, e.g., AcVDVKD-CHO [casp-2 pKi: 7.63, casp-3 pKi: 6.91] and AcVDV(Dab)D-CHO [casp-2 pKi: 7.26; casp-3 pKi: 5.82], turned out to be more selective toward casp-2 over casp-3 [119].

It is worth emphasizing that the application of the computational tools streamlines the process of lead identification and allows for a more efficient and targeted approach. Despite significant progress in the preclinical field, translating these findings into safe and effective therapies for individuals suffering from Alzheimer’s disease remains a significant challenge. Further research and clinical trials are necessary to better understand the underlying mechanisms of this disorder and to develop more efficacious treatments.

### Clinical Studies on Caspase Inhibitors

**Fig. 8:**
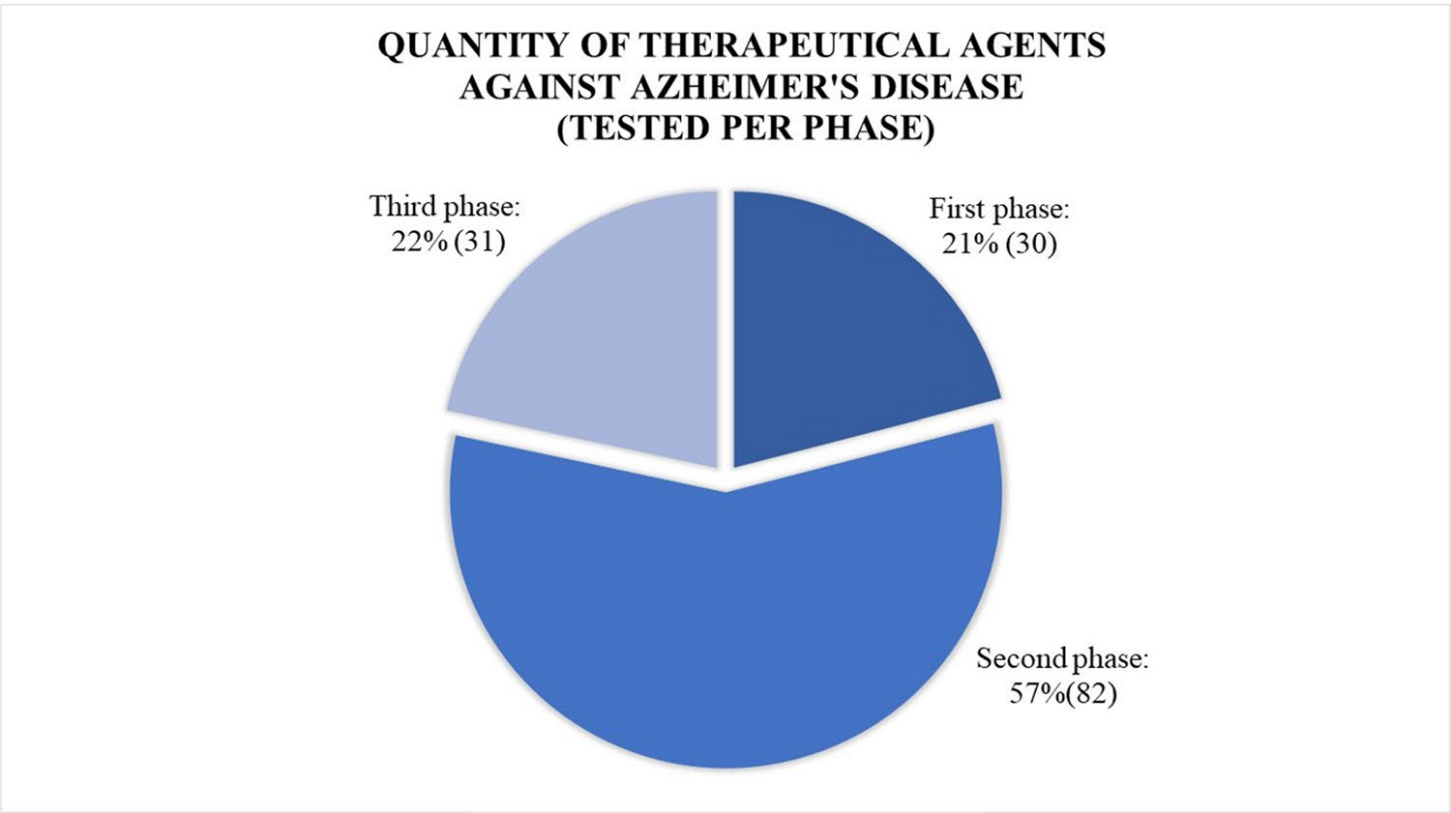
Distribution of novel therapeutical agents tested for their effectiveness and safety in patients suffering from Alzheimer’s disease

The search for effective treatments for Alzheimer’s disease is a top priority for many medicinal chemistry researchers. While information on promising compounds is regularly published, only a small number progress to the final marketing phase as shown in Fig. 8. As of 2022, 143 agents with various mechanisms of action have been submitted for 172 clinical trials, reflecting an increase of 17 compared to 2021 data. This significant increase may be attributed in part to improved search techniques and increased knowledge about the disease and ideal drug candidate characteristics [120].

Currently, there are no caspase inhibitors approved by the Food and Drug Administration or European Medicines Agency for the treatment of Alzheimer’s disease. However, some promising agents, such as Emricasan and CEP-1347, have shown improvement in cognitive function and reduction in neuronal death in preclinical models of Alzheimer’s disease. These agents were evaluated in clinical trials for other diseases but have also been proposed as potential treatments for Alzheimer’s disease.

Emricasan is a caspase inhibitor evaluated in clinical trials for potential application in alcoholic liver disease [121]. More recently, its caspase-1 inhibitory properties were evaluated in another trial, aiming to determine its usefulness for the treatment of activated inflammasome in mild COVID-19 [122]. Preclinical studies have shown its ability to reduce neuronal death and improve cognitive functions in models of Alzheimer’s disease. It has also been demonstrated to reduce inflammation and oxidative stress in these models. Its activity profile makes it a promising agent worth further investigation in terms of inflammasome modulation [116]. CEP-1347 is a small molecule that inhibits the activity of several different kinases, including caspase-3. It has been shown to improve cognitive function and reduce neuronal death in preclinical models of Alzheimer’s disease. Currently, CEP-1347 is being evaluated in a phase 2 clinical trial for the treatment of Parkinson’s disease, but it has also been proposed as a potential treatment for Alzheimer’s disease [123].

In-depth evaluation of various caspase inhibitors has contributed to our understanding of cell death mechanisms and inflammation. Despite the promising results obtained in preclinical studies, the clinical development of caspase inhibitors for the treatment of Alzheimer’s disease has been challenging. One of the main issues is the difficulty in targeting caspases without affecting other important cellular processes regulated by these enzymes. Additionally, the blood–brain barrier, which protects the brain from potentially harmful agents, can limit the application and effectiveness of promising molecules. The ongoing research in this direction provides hope for the discovery of new and effective caspase inhibitors that will help treat patients with Alzheimer’s disease. Another challenge is identifying which caspases are crucial in AD pathology. Pan-caspase inhibitors may initially seem like an ideal solution, but they can strongly inhibit the ability of cells to undergo apoptosis, which is a physiological process necessary for the proper functioning of the human body. On the other hand, drugs that specifically inhibit the final stages of apoptosis by targeting caspase-3 or those that inhibit caspase-8 and caspase-1 seem more promising. Caspase-8 is involved in both Aβ metabolism and the activation of the extrinsic pathway of apoptosis, suggesting the crucial role of these actions in modulating AD through caspases. However, inhibiting caspase-8 may lead to necroptosis, especially in pro-inflammatory conditions, which would further increase neuroinflammation. On the other hand, caspase-1 inhibition may ameliorate pyroptosis, potentially preventing the side effects associated with caspase-8 inhibition.

## Conclusions and Perspectives

Due to the aging of the population, neurodegenerative diseases, such as Alzheimer’s disease, have become a serious medical problem nowadays. Although visible symptoms include cognitive dysfunction, memory loss, etc., at the brain level, loss of neurons is observed, which is probably the cause of the cognitive dysfunction. However, the exact cause of neuronal loss remains unclear. Different hypotheses have been developed to explain this phenomenon, but the most important and widely accepted ones are the β-amyloid, Tau protein, and mitochondrial failure hypotheses.

In Alzheimer’s disease, insoluble forms of amyloid β aggregate and deposit in the form of plaques in extracellular spaces and the walls of blood vessels. Similarly, the Tau microtubule protein aggregates into neurofibrillary tangles in neurons. The pathogenesis of AD begins with the accumulation of Aβ in the cortex or neurofibrillary tangles, which may precede the initial symptoms by many years. Additionally, as neurons demand a large amount of energy, they are especially sensitive to any problems with mitochondria. Therefore, according to this theory, mitochondrial damage is not a result but a cause of neurodegeneration. However, the activation of caspases is a common point in each of these theories, as ultimately, their activation leads to cell death. Although cell death can also occur via caspase-independent pathways, a body of evidence suggests that in AD, pyroptosis and apoptosis are the dominant mechanisms, and these types of cell death are entirely caspase-dependent. Still, only the mitochondrial theory reduces caspases to a role in the final stage of cell death. In this theory, the release of ROS from the mitochondria, leading to DNA damage, as well as the direct release of pro-apoptotic proteins from the mitochondria, activates the intrinsic pathway, resulting in the activation of caspase 9. Moreover, ROS, by damaging proteins, activate ER stress, which results in the activation of caspase 2.

Nevertheless, in the case of β-amyloid and Tau protein hypotheses, the action of caspases has two faces. In the first, similar to the mitochondrial theory, caspases are responsible for cell death because both amyloid and Tau are highly toxic. As a result, these proteins can activate caspases in various ways. By interacting with death receptors, they can activate the extrinsic pathway, which activates caspase 8. Their accumulation in the ER can cause ER stress, activating caspase 2, and by interacting with mitochondrial proteins, they induce the intrinsic pathway and caspase 9. However, genetic engineering studies suggest that activation of caspase 8 plays the most important role in inducing apoptosis in AD. Moreover, Aβ and Tau can also induce necroptosis and pyroptosis. Pyroptosis, associated with the activation of caspase 1, seems to be important in the pathogenesis of AD because not only is it intensified but, contrary to apoptosis, it significantly increases inflammation, which, in turn, increases the activation of caspases (especially caspase 8, as death receptors can be activated by the pro-inflammatory TNFα).

On the other hand, according to these theories, caspases, especially caspases 8 and 3, also play a role in the initial stages of AD development because, as proteolytic enzymes, they cleave amyloid precursor protein and Tau protein into fragments that easily aggregate. A positive feedback loop is visible here, as the accumulation of these proteins, on the other hand, increases the activation of caspases, leading to further and stronger accumulation of these proteins and apoptosis, if the activation of caspases is sufficiently increased.

Given the significant role caspases seem to play in Alzheimer’s disease, it is not surprising that caspase inhibitors are being considered and tested as drugs against Alzheimer’s disease. However, this is associated with significant problems. Firstly, the blood–brain barrier inhibits the penetration of drugs into the brain, and secondly, the action of caspases is necessary for the functioning of the human body, so high doses or more powerful drugs seem to be a dead end. Nevertheless, caspase inhibitors with high therapeutic potential are still being tested, and the most promising ones seem to be those that interact specifically with caspases 3, 8, or 1. Inhibition of caspases 3 and 8 blocks the extrinsic apoptosis pathway, which seems to be a crucial pathway in AD. Additionally, as these caspases play a role in Aβ and Tau protein processing, their inhibition may counteract AD at an earlier stage. On the other hand, caspase 1 inhibitors counteract pyroptosis, which not only decreases neuronal loss but also neuroinflammation.

## Data Availability

Not applicable.
